# On Some Topological Indices Defined via the Modified Sombor Matrix

**DOI:** 10.3390/molecules27196772

**Published:** 2022-10-10

**Authors:** Xuewu Zuo, Bilal Ahmad Rather, Muhammad Imran, Akbar Ali

**Affiliations:** 1 Department of Common Course, Anhui Xinhua University, Hefei 230088, China; 2 Department of Mathematical Sciences, College of Science, United Arab Emirates University, Al Ain, Abu Dhabi 15551, United Arab Emirates; 3 Department of Mathematics, Faculty of Science, University of Ha’il, Ha’il P.O. Box 2240, Saudi Arabia

**Keywords:** adjacency matrix, Sombor index, modified Sombor matrix, modified Sombor energy, correlation

## Abstract

Let *G* be a simple graph with the vertex set $V=\{v_{1},\ldots ,v_{n}\}$ and denote by $d_{v_{i}}$ the degree of the vertex $v_{i}$. The modified Sombor index of *G* is the addition of the numbers ${\left(d_{v_{i}}^{2}+d_{v_{j}}^{2}\right)}^{-1/2}$ over all of the edges $v_{i}v_{j}$ of *G*. The modified Sombor matrix $A_{MS}\left(G\right)$ of *G* is the *n* by *n* matrix such that its (i,j)-entry is equal to ${\left(d_{v_{i}}^{2}+d_{v_{j}}^{2}\right)}^{-1/2}$ when $v_{i}$ and $v_{j}$ are adjacent and 0 otherwise. The modified Sombor spectral radius of *G* is the largest number among all of the eigenvalues of $A_{MS}\left(G\right)$. The sum of the absolute eigenvalues of $A_{MS}\left(G\right)$ is known as the modified Sombor energy of *G*. Two graphs with the same modified Sombor energy are referred to as modified Sombor equienergetic graphs. In this article, several bounds for the modified Sombor index, the modified Sombor spectral radius, and the modified Sombor energy are found, and the corresponding extremal graphs are characterized. By using computer programs (Mathematica and AutographiX), it is found that there exists only one pair of the modified Sombor equienergetic chemical graphs of an order of at most seven. It is proven that the modified Sombor energy of every regular, complete multipartite graph is $\sqrt{2}$; this result gives a large class of the modified Sombor equienergetic graphs. The (linear, logarithmic, and quadratic) regression analyses of the modified Sombor index and the modified Sombor energy together with their classical versions are also performed for the boiling points of the chemical graphs of an order of at most seven.

## 1. Introduction

We consider only simple and undirected graphs. The graph theoretical terminologies used in this paper, without defining them here, can be found in the book [1]. A graph of the order *n* with a vertex set V(G) and an edge set E(G) is denoted by G(V,E), or simply by *G*, where $V\left(G\right)=\left\{v_{1},v_{2},\ldots ,v_{n}\right\}$. The cardinality of E(G) is the size *m* of *G*. The *degree* of a vertex *v* in *G* is the number of edges incident with *v* and is denoted by $d_{v}$. A *regular* graph is one in which each vertex has the same degree. The maximum and minimum degrees of *G* are denoted by Δ and δ, respectively. A graph of the order *n* is also known as an *n*-vertex graph. $K_{n},\;K_{a,b},$ and $P_{n}$ denote the *n*-vertex complete graph, the (a+b)-vertex complete bipartite graph, and the *n*-vertex path graph, respectively. Moreover, denote the complete multipartite graph by $K_{n_{1},n_{2},\ldots ,n_{t}}$, where t≥3, and denote the complete split graph with the clique size ω and the independence number n−ω by CS(ω,n−ω). Let $S_{n}^{+}$ be the graph formed by adding an edge to the star $K_{1,n-1}$.

By a topological index, we mean a numerical quantity TI calculated from a graph such that TI remains unchanged under graph isomorphism. Topological indices have several uses in theoretical chemistry, especially in quantitative structure–activity relationship and quantitative structure–property relationship studies [2]. For a graph G, its degree-based topological indices ϕ [3,4,5] of the following form are known as bond incident degree indices [6]:

$$\phi \left(G\right)=\sum_{uv\in E(G)}\phi_{d_{u},d_{v}},$$

where $\phi_{d_{u},d_{v}}$ is a function with the property $\phi_{d_{u},d_{v}}=\phi_{d_{v},d_{u}}$. For particular choices of $\phi_{d_{u},d_{v}}$, we obtain existing topological indices; for example, the arithmetic–geometric index [7] is obtained when $\phi_{d_{u},d_{v}}=\left(d_{u}+d_{v}\right){\left(4d_{u}d_{v}\right)}^{-1/2}$, the general Randić index [8] is recovered if $\phi_{d_{u},d_{v}}={\left(d_{u}d_{v}\right)}^{\alpha },$ (for α=−1/2, we obtain the classical Randić index *R* [9]), and the general Sombor index is deduced when $\phi_{d_{u},d_{v}}={\left(d_{u}^{2}+d_{v}^{2}\right)}^{\alpha }$. From the general Sombor index, we obtain the recently introduced Sombor (SO) index [10] and the modified Sombor index ${}^{m}SO$ [11] by using α=1/2 and α=−1/2, respectively.

The basic properties of the Sombor index were given by Gutman [10]. Das et al. [12] presented novel bounds for the Sombor index and gave its relations with several other topological indices, such as the Zagreb indices. Cruz et al. [13] investigated the Sombor index for chemical graphs and characterized extremal graphs from the classes of chemical graphs, chemical trees, and hexagonal systems, with respect to this index. The chemical applicability of the Sombor index was investigated in [14,15]. Kulli and Gutman initiated the study of the modified Sombor index and gave its basic properties. Later, Huang and Liu [16] obtained several interesting properties and bounds of the modified Sombor index, and they found its relations with some other topological indices, such as the Randić index, the Harmonic index, the sum-connectivity index, and the geometric–arithmetic index.

The general adjacency matrix (for example, see [4]) associated with ϕ of *G* is a real symmetric matrix, defined by

(1) $$A_{\phi }\left(G\right)={\left(a_{\phi }\right)}_{ij}=\left\{\begin{array}{cc} \phi_{d_{u},d_{v}} & \mathrm{if}\;uv\in E(G)\\ 0 & \mathrm{otherwise}. \end{array}\right.$$

The set of all eigenvalues of $A_{\phi }\left(G\right)$ is known as the *general adjacency spectrum* of *G* and is denoted by $\lambda_{1}\left(A_{\phi }\left(G\right)\right),\ldots ,\lambda_{n}\left(A_{\phi }\left(G\right)\right),$ indexed in a non-increasing order, where $\lambda_{1}\left(A_{\phi }\left(G\right)\right)$ is known as the general adjacency spectral radius of G. If *G* is a connected non-trivial graph and $\phi_{d_{u},d_{v}}>0$ for every edge uv∈E(G), then by the Perron–Frobenius theorem, $\lambda_{1}\left(A_{\phi }\left(G\right)\right)$ is unique, and its associated eigenvector has positive components. Moreover, in this case, the inequality $|\lambda_{i}\left(A_{\phi }\left(G\right)\right)|\leq \lambda_{1}\left(A_{\phi }\left(G\right)\right)$ holds for every i∈{2,…,n−1,n}. The energy of the graph *G* associated with the topological index ϕ is defined [17] as

$$E_{\phi }\left(G\right)=\sum_{i=1}^{n}\left|\lambda_{i}\left(A_{\phi }\left(G\right)\right)\right|.$$

If $\phi_{d_{u},d_{v}}=1$ for every edge uv∈E(G), then $A_{\phi }\left(G\right)$ coincides with the much-studied adjacency matrix A(G), and $E_{\phi }\left(G\right)$ is the classical graph energy [18] defined as $E\left(G\right)=\sum_{i=1}^{n}\left|\lambda_{i}\right|$, where $\lambda_{1},\ldots ,\lambda_{n}$ are the eigenvalues of A(G), and the multiset consisting of these eigenvalues is known as the *spectrum* of *G*. The graph energy E(G) has its origin in theoretical chemistry and helps in approximating the π-electron energy of unsaturated hydrocarbons. There is a wealth of literature about graph energy and its related topics (for examples, see [19,20,21,22,23,24,25]).

If we take $\phi_{d_{u},d_{v}}=\sqrt{d_{u}^{2}+d_{v}^{2}}$ in (1), the we obtain the Sombor matrix

$$A_{S}\left(G\right)=\left\{\begin{array}{cc} \sqrt{d_{u}^{2}+d_{v}^{2}} & \mathrm{if}\;uv\in E(G)\\ 0 & \mathrm{otherwise}. \end{array}\right.$$

We denote each eigenvalue of $A_{S}\left(G\right)$ by $\mu_{i}$ and order them as $\mu_{1}\geq \cdots \geq \mu_{n}$. The multiset of all eigenvalues of $A_{S}\left(G\right)$ is known as the *Sombor spectrum* of G. The Sombor energy of *G* is defined by

$$E_{SO}\left(G\right)=\sum_{i=1}^{n}\left|\mu_{i}\right|.$$

Two graphs with the same modified Sombor energy are referred to as *modified Sombor equienergetic graphs*. Various papers on the spectral properties of the Sombor matrix, involving Sombor eigenvalues, the Sombor spectral radius, Sombor energy, the Sombor Estrada index, the relation of energy with Sombor energy, and the Sombor index, have recently been published (for examples, see [22,26,27,28,29,30,31,32,33,34,35]).

The substitution $\phi_{d_{u},d_{v}}={\left(d_{u}^{2}+d_{v}^{2}\right)}^{-1/2}$ in (1) yields the modified Sombor matrix

$$A_{MS}\left(G\right)=\left\{\begin{array}{cc} \frac{1}{\sqrt{d_{u}^{2}+d_{v}^{2}}} & \mathrm{if}\;uv\in E(G)\\ 0 & \mathrm{otherwise}. \end{array}\right.$$

The multiset consisting of all of the eigenvalues $\rho_{1},\ldots ,\rho_{n}$ of $A_{MS}\left(G\right)$ is called the *modified Sombor spectrum* of *G*. We assume that $\rho_{1}\geq \cdots \geq \rho_{n},$ where $\rho_{1}$ is called the *modified Sombor spectral* radius of *G*. The modified Sombor energy [16] is defined by

$$E_{MS}\left(G\right)=\sum_{i=1}^{n}\left|\rho_{i}\right|.$$

Various properties concerning the modified Sombor matrix can be found in [16].

The chemical applicability of the Sombor indices, such as the predictive and discriminative potentials, was examined by [15]. The Sombor index, the reduced Sombor index, and the average Sombor index were used to model the entropy and enthalpy of vaporization of alkanes. Some linear models that use one of these indices as the only predictor showed satisfactory predictive potential. The performance of these models was improved with the introduction of other topological indices, such as the first Zagreb index as a second predictor. Among these three topological molecular descriptors, the reduced Sombor index showed the best performance. The results of testing the predictive potential of the Sombor indices indicate that these descriptors may be successfully applied to modeling the thermodynamic properties of compounds.

The bond incident degree indices and their corresponding matrices (weighted adjacency matrices) have their own significance. Some notable points regarding some weighted adjacency matrices are below:

- The classical graph energy cannot be an odd integer (see [36]).
- The arithmetic–geometric energy can be any positive integer greater than one (see [37]).
- The modified Sombor energy of every regular complete multipartite is constant and equals $\sqrt{2}$ (see Corollary 1).
- The modified Sombor spectral radius of every regular graph is constant and equals $\frac{1}{\sqrt{2}}$ (see Proposition 2).

The remainder of this paper is organized as follows: In Section 2, we establish bounds on the modified Sombor index, the modified Sombor spectral radius, and the modified Sombor energy and determine all of the graphs that attain these bounds. In Section 3, by using computer programs (Mathematica and AutographiX), we find that there exists only one pair of the modified Sombor equienergetic chemical graphs of an order of at most seven. The (linear, logarithmic, and quadratic) regression analyses of the modified Sombor index and the modified Sombor energy together with their classical versions are also performed in Section 3 for the boiling points of chemical graphs of an order of at most seven.

## 2. Results Concerning the Modified Sombor Matrix

In this section, we give bounds on the modified Sombor index, the modified Sombor spectral radius, and the modified Sombor energy, and we characterize the graphs that attain these bounds.

Let $\sigma_{1},\ldots ,\sigma_{n}$ be singular values of a matrix *M*. The *Frobenius norm* of *M* is defined by

$${∥M∥}_{F}^{2}=\sigma_{1}^{2}+\sigma_{2}^{2}+\cdots +\sigma_{n}^{2}.$$

Similarly, the Frobenius norm (see [16]) of the modified Sombor matrix $A_{MS}\left(G\right)$ is

$$\rho_{1}^{2}+\rho_{2}^{2}+\cdots +\rho_{n}^{2}={∥A_{MS}\left(G\right)∥}_{F}^{2}=2B=Tr\left(A_{MS}^{2}\left(G\right)\right),$$

where

(2) $$B=\sum_{v_{i}v_{j}\in E\left(G\right)}\frac{1}{d_{v_{i}}^{2}+d_{v_{j}}^{2}}$$

and “Tr” denotes the trace of a matrix. We note that the modified Sombor index can be expressed as a quadratic form of the modified Sombor matrix:

$${}^{m}SO\left(G\right)=\frac{1}{2}\left(J^{T}A_{MS}\left(G\right)J\right),$$

where *J* is a matrix of all ones. Moreover, according to the Rayleigh–Ritz theorem [19], for a non-zero vector *X*, we have

(3) $$\rho_{1}\left(G\right)=\max_{X\neq 0}\frac{X^{T}A_{MS}\left(G\right)X}{X^{T}X}\geq \frac{J^{T}A_{MS}\left(G\right)J}{J^{T}J}=\frac{2{(}^{m}SO\left(G\right))}{n}.$$

If *G* is an *r*-regular graph, then

$$A_{MS}\left(G\right)=\frac{1}{r\sqrt{2}}A\left(G\right),$$

and it is well known that $\lambda_{1}\leq \Delta$ (see Propositions 1.1.1 and 1.1.2 [19]) with equality if and only if *G* is regular. Thus, for regular graphs, we have

$$\rho_{1}=\frac{1}{\sqrt{2}}\;\mathrm{and}\;\frac{2{(}^{m}SO\left(G\right))}{n}=\frac{2m}{r\sqrt{2}n}=\frac{1}{\sqrt{2}},$$

because m=nr/2. From these observations, the next two results follow:

**Proposition** **1.** *If G is a connected non-trivial graph of the order n, then*

$${}^{m}SO\left(G\right)\leq \frac{n\rho_{1}}{2},$$

*with equality holding if and only G is regular.*

**Proposition** **2.** *The modified Sombor spectral radius of every regular non-trivial graph is $\frac{1}{\sqrt{2}}.$*

The number of edges adjacent to an edge uv of a graph is called the edge degree of uv. Following Simić and Stanić [38], we say that a graph is *edge-regular* (same terminology is also used for a particular type of regular graph; for example, see [39]) if all its edges have the same edge degree. The following result gives an upper bound for ${}^{m}SO\left(G\right)$ in terms of the size *m* and the Frobenius norm of $A_{MS}\left(G\right).$

**Proposition** **3.** *If G is a connected non-trivial graph of size m, then*

$${}^{m}SO\left(G\right)\leq \sqrt{m\cdot B},$$

*with equality holding if and only G is edge-regular, where B is defined via (2).*

**Proof.**  By using the Cauchy–Schwarz inequality, we have

$$\begin{array}{c} \sum_{uv\in E(G)}\frac{1}{\sqrt{d_{u}^{2}+d_{v}^{2}}}\leq \sqrt{m\sum_{uv\in E(G)}\frac{1}{d_{u}^{2}+d_{v}^{2}}}, \end{array}$$

with equality holding if and only if there exists a constant *c* such that the equation $d_{u}^{2}+d_{v}^{2}=c$ holds for every edge uv∈E(G). If w∈V(G) is a vertex of at least two degrees, and $w_{1},w_{2}\in V\left(G\right)$ are two of its neighbors, then the equation $d_{w_{1}}^{2}+d_{w}^{2}=d_{w_{2}}^{2}+d_{w}^{2}$ holds if and only if $d_{w_{1}}=d_{w_{2}}$. Thus, the equation $d_{u}^{2}+d_{v}^{2}=c$ holds for every edge uv∈E(G) if and only if all of the neighbors of every vertex of *G* have the same degree; that is, *G* is an edge-regular graph. □

**Figure 1:**
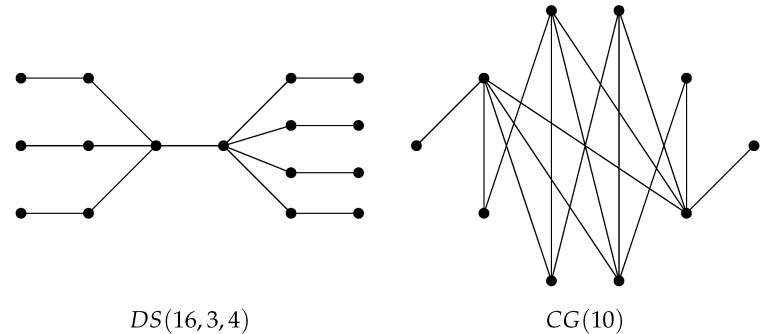
Double star-type graph DS(16, 3, 4) on 16 vertices and the chain graph CG(10) on 10 vertices.

The *double star-type* graph DS(n, a, b) is a tree obtained from the two-vertex complete graph $K_{2}$ by attaching a pendent vertex of each of the *a* copies of the three-vertex path graph $P_{3}$ to one end-vertex of $K_{2}$ and attaching a pendent vertex of each of the *b* copies of $P_{3}$ to its other end-vertex; see Figure 1 for the graph DS(16, 3, 4). A graph of the order *n* is said to be a *chain graph* CG(n) if it is bipartite and the neighborhoods of the vertices in each color class form a chain with respect to set inclusion (see Figure 1).

Table 1 presents the numerical calculations of the modified Sombor index bounds obtained in Propositions 1 and 3 and the results obtained by Huang and Liu [16].

The following result gives a lower bound and an upper bound for the modified Sombor spectral radius.

**Proposition** **4.** *Let B be the topological index defined via (2). If G is a non-trivial graph of the order n, then*

(4) $$\sqrt{\frac{2B}{n}}\leq \rho_{1}\leq \sqrt{\frac{2B(n-1)}{n}},$$

*where the left equality sign holds if and only if $|\rho_{1}\left|=\right|\rho_{2}\left|=\ldots =\right|\rho_{n}|.$ If G is connected, then the right equality in (4) holds if and only if $G≅K_{n}$.*

**Proof.**  The right-handed inequality in (4) has already been derived in Theorem 3.4 [16] but without identifying the graphs that attain equality. For the sake of completeness, we first give the proof of this inequality and then characterize the connected graphs that attain its equality sign. Applying the Cauchy–Schwarz inequality to the vector ($\rho_{2},\;\rho_{3},\;\ldots ,\;\rho_{n}$), we have

(5) $$\rho_{1}^{2}=2B-\sum_{i=2}^{n}\rho_{i}^{2}\leq 2B-\frac{1}{n-1}{\left(\sum_{i=2}^{n}\rho_{i}\right)}^{2}=2B-\frac{1}{n-1}\rho_{1}^{2},$$

which implies that

(6) $$\rho_{1}\leq \sqrt{\frac{2B(n-1)}{n}}.$$

Suppose equality holds in (6). Then, equality holds in (5), which is possible if and only if $\rho_{2}=\rho_{3}=\cdots =\rho_{n}.$ That is, *G* has two distinct modified Sombor eigenvalues $\rho_{1}$ and $\rho_{2}$. According to the proof of Proposition 1.3.3 of [40], the diameter of *G* must be one, which implies that *G* is $K_{n}$. Conversely, if $G≅K_{n},$ then the modified Sombor spectrum of *G* is

$$\left\{\frac{1}{\sqrt{2}},{\left(\frac{-1}{\left(n-1\right)\sqrt{2}}\right)}^{\left[n-1\right]}\right\}\;\mathrm{and}\;B=\frac{n}{4(n-1)}.$$

Thus,

$$\rho_{1}=\sqrt{\frac{2B(n-1)}{n}}=\sqrt{\frac{1}{2}\cdot \frac{n}{n-1}\cdot \frac{n-1}{n}}=\frac{1}{\sqrt{2}}.$$

Recall that $\rho_{1}^{2}+\rho_{2}^{2}+\cdots +\rho_{n}^{2}=2B,$ so we obtain $2B\leq \rho_{1}^{2}+\rho_{1}^{2}+\cdots +\rho_{1}^{2},$ which gives

$$\rho_{1}\geq \sqrt{\frac{2B}{n}}$$

with equality holding if and only if $|\rho_{1}\left|=\right|\rho_{2}\left|=\cdots =\right|\rho_{n}|.$ □

From the equality case of the right-handed inequality of Proposition 4, Proposition 1.3.3 of [40], and Theorem 2.1 of [41], the next proposition follows:

**Proposition** **5.** *Let G be a connected non-trivial graph. The graph G has only two distinct modified Sombor eigenvalues if and only if G is the complete graph.*

The topological index *B* defined via (2) is repeatedly used in our results. We now establish some bounds on it and characterize the corresponding extremal graphs.

**Proposition** **6.** *Let G be a connected non-trivial graph.*

(i) *If G has a size m, maximum degree* Δ, *and minimum degree* δ, *then*
  $$\frac{m}{2\Delta^{2}}\leq B\leq \frac{m}{2\delta^{2}},$$
*where either of the two equalities holds if and only if G is regular.*
(ii) *If G has the order n and the modified Sombor spectral radius $\rho_{1}$, then*
$$B\leq \frac{n\rho_{1}^{2}}{2(n-1)},$$
*with equality holding if and only if $G≅K_{n}.$*
(iii) *For t≥1, if G is bipartite with exactly t positive modified Sombor eigenvalues, then*
$$B\geq t\rho_{1}^{2},$$
*with equality holding if and only if G is the complete bipartite graph.*

**Proof.**  Since $\delta \leq d_{v}\leq \Delta$ for every vertex v∈V(G), Part (i) follows directly from the definition of *B*. Moreover, Part (ii) is a direct consequence of Proposition 4. In what follows, we prove Part (iii). Note that $\rho_{1}^{2}+\rho_{2}^{2}+\cdots +\rho_{n}^{2}=2B$. Since *G* has exactly *t* positive modified Sombor eigenvalues, and its modified Sombor is symmetric toward the origin, we obtain

$$2\left(\rho_{1}^{2}+\rho_{2}^{2}+\ldots +\rho_{t}^{2}\right)=2B,$$

which implies that $B\geq t\rho_{1}^{2}$, with equality if and only if t=1; by Proposition 7, *G* is the complete bipartite graph. □

Clearly, it holds that $A_{MS}\left(G\right)=\frac{1}{r\sqrt{2}}A\left(G\right)$ for an *r*-regular graph *G*. Thus, for regular graphs, we have

$$E_{MS}\left(G\right)=\frac{1}{r\sqrt{2}}E\left(G\right)=\frac{1}{2r^{2}}E_{SO}\left(G\right).$$

Moreover, from the definition of $E_{MS}\left(G\right)$, it follows that

(7) $$E_{MS}\left(G\right)=\sum_{i=1}^{n}\left|\rho_{i}\right|=2\sum_{\begin{array}{c} i=1\\ \rho_{i}\geq 0 \end{array}}^{n}\rho_{i}\geq 2\rho_{1},$$

where the right-handed equality sign holds if and only if *G* has only one positive modified Sombor eigenvalue. From (7) and any lower bound on $\rho_{1}$, we obtain another lower bound on $E_{MS}\left(G\right)$. For example, we derive two such lower bounds on $E_{MS}\left(G\right)$ in the following:

From Proposition 1, it follows that

$$\rho_{1}\geq \frac{2{(}^{m}SO\left(G\right))}{n},$$

with equality if and only if *G* is regular. Moreover, according to Proposition 4, we have

$$\rho_{1}\geq \sqrt{\frac{2B}{n}},$$

with equality if and only if $|\rho_{1}\left|=\right|\rho_{2}\left|=\cdots =\right|\rho_{n}|$. Thus, from these two lower bounds on $\rho_{1}$ and from (7), the next two lower bounds on $E_{MS}$ follow

(8) $$\begin{array}{cc} E_{MS}\left(G\right)\geq & \;\frac{4{(}^{m}SO\left(G\right))}{n}, \end{array}$$

(9) $$\begin{array}{cc} E_{MS}\left(G\right)\geq & \;2\sqrt{\frac{2B}{n}}. \end{array}$$

Equality occurs in (8) if and only if *G* is regular and has exactly one positive modified Sombor eigenvalue. Recall that, for regular graphs, we have =$A_{MS}\left(G\right)=\frac{1}{r\sqrt{2}}A\left(G\right)$ and that the adjacency matrix A(G) has only one positive eigenvalue if and only if *G* is a complete multipartite graph (see Smith [42]). Thus, equality holds in (8) if and only if *G* is a regular complete multipartite graph. Moreover, equality holds in () if and only *G* has only one positive modified Sombor eigenvalue and $|\rho_{1}\left|=\right|\rho_{2}\left|=\cdots =\right|\rho_{n}|$; which means that *G* is $\frac{n}{2}K_{2}$ when *G* is disconnected, and *G* is $K_{2}$ when *G* is connected. We summarize these observations in the following result.

**Theorem** **1.** *Let G be a non-trivial graph of order n.*

(i) *It holds that*
$$E_{MS}\left(G\right)\geq \frac{4{(}^{m}SO\left(G\right))}{n},$$
  *with equality if and only if G is a regular complete multipartite graph.*
(ii) *It holds that*
$$E_{MS}\left(G\right)\geq 2\sqrt{\frac{2B}{n}},$$
  *with equality if and only if G is $\frac{n}{2}K_{2}$ when G is disconnected, and G is $K_{2}$ when G is connected.*

Next, we have an immediate consequence of Theorem 1, which states that the modified Sombor energy of the regular complete multipartite graphs is constant (which implies that the equation $E_{MS}\left(G_{1}\right)=E_{MS}\left(G_{2}\right)$ holds for every pair of nonisomorphic regular complete multipartite graphs $G_{1}$ and $G_{2}$ of the same order).

**Corollary** **1.** *If G is a regular complete multipartite graph, then*

$$E_{MS}\left(G\right)=\sqrt{2}.$$

**Remark** **1.** *If G is a connected graph, then*

$$\begin{array}{cc} \frac{2}{n}\left({\;}^{m}SO\left(G\right)\right) & =\frac{2}{n}\sum_{uv\in E}\frac{1}{\sqrt{d_{u}^{2}+d_{v}^{2}}}\\ \; & >\frac{2}{n}\sum_{uv\in E}\frac{1}{d_{u}^{2}+d_{v}^{2}}=\frac{2B}{n}\geq \sqrt{\frac{2B}{n}}. \end{array}$$

*Thus, for connected graphs, the lower bound on $\rho_{1}$ given in Proposition 1 is better than the one given in Proposition 4.*

Next, we give the McClelland- and the Koolen–Moulton-type bounds for the modified Sombor energy.

**Theorem** **2.** (i) *If G is a graph of the order n, and B is the topological index defined via (2), then*

$$E_{MS}\left(G\right)\leq \frac{2{(}^{m}SO\left(G\right))}{n}+\sqrt{\left(n-1\right)\left(2B-{\left(\frac{2{(}^{m}SO\left(G\right))}{n}\right)}^{2}\right)}.$$

*If G is connected, then equality holds if and only if G is either $K_{n}$ or G has three distinct modified Sombor eigenvalues: $\rho_{1}=\frac{2{(}^{m}SO\left(G\right))}{n}$ and two others with equal absolute values*

$$|\rho_{2}\left|=\right|\rho_{3}\left|=\ldots =\right|\rho_{n}|=\sqrt{\frac{1}{\left(n-1\right)}(2B-{\left(\frac{2{(}^{m}SO\left(G\right))}{n}\right)}^{2}}).$$

(ii) *If G is without any isolated vertex, then*

$$E_{MS}\left(G\right)\leq \sqrt{2nB},$$

*with equality holding if and only if $G≅\frac{n}{2}K_{2}.$*

**Proof.**  By applying the Cauchy–Schwartz inequality and then using the fact that $\sum_{i=1}^{n}\rho_{i}^{2}=2B$, we have

$$\begin{array}{cc} E_{MS}\left(G\right) & =\rho_{1}+\sum_{i=2}^{n}\left|\rho_{i}\right|\\ \; & \leq \rho_{1}+\sqrt{\left(n-1\right)\sum_{i=2}^{n}\rho_{i}^{2}}=\rho_{1}+\sqrt{\left(n-1\right)\left(2B-\rho_{1}^{2}\right)}, \end{array}$$

where the inequality sign becomes an equality sign if and only if

$$|\rho_{2}\left|=\right|\rho_{3}\left|=\ldots =\right|\rho_{n}|=\sqrt{\frac{2B-\rho_{1}^{2}}{n-1}}.$$

Clearly, the function *F* defined by

(10) $$F\left(x\right)=x+\sqrt{\left(n-1\right)\left(2B-x^{2}\right)}$$

is decreasing for *x* in the interval $\left[\sqrt{\frac{2B}{n}},\sqrt{2B}\right]$. From Proposition 1, it follows that

$$\rho_{1}\geq \frac{2{(}^{m}SO\left(G\right))}{n}$$

with equality if and only if $G≅K_{n}$. Moreover, according to Remark 1,

$$\frac{2{(}^{m}SO\left(G\right))}{n}\geq \sqrt{\frac{2B}{n}}.$$

Thus,

$$F\left(\rho_{1}\right)\leq F\left(\frac{2{(}^{m}SO\left(G\right))}{n}\right)$$

and hence

(11) $$E_{MS}\left(G\right)\leq \frac{2{(}^{m}SO\left(G\right))}{n}+\sqrt{\left(n-1\right)\left(2B-{\left(\frac{2{(}^{m}SO\left(G\right))}{n}\right)}^{2}\right)}.$$

Equality holds in (11) if and only if all above equalities hold, i.e., *G* is regular with the modified Sombor spectrum satisfying: $\rho_{1}=\frac{2{(}^{m}SO\left(G\right))}{n}$ and $|\rho_{2}\left|=\right|\rho_{3}\left|=\cdots =\right|\rho_{n}|.$ One possibility is that *G* has two distinct modified Sombor eigenvalues and, according to Proposition 5, *G* is the complete graph. Conversely, for $G≅K_{n}$, we have ${}^{m}SO\left(K_{n}\right)=\frac{m}{\left(n-1\right)\sqrt{2}}=\frac{n}{2\sqrt{2}}$, $B=\frac{n}{4(n-1)}$, and

$$\begin{array}{cc} \; & \frac{2{(}^{m}SO\left(G\right))}{n}+\sqrt{\left(n-1\right)\left(2B-{\left(\frac{2{(}^{m}SO\left(G\right))}{n}\right)}^{2}\right)}\\ & =\frac{1}{\sqrt{2}}+\sqrt{\left(n-1\right)\left(\frac{n}{2(n-1)}-\frac{1}{2}\right)}\\ \; & =\frac{2}{\sqrt{2}}=2\rho_{1}\left(K_{n}\right)=E_{MS}\left(K_{n}\right). \end{array}$$

Therefore, equality holds if and only if either $G≅K_{n}$ or *G* has three distinct modified Sombor eigenvalues: $\rho_{1}=\frac{2{(}^{m}SO\left(G\right))}{n}$ and the remaining modified Sombor eigenvalues are equal in absolute value

$$|\rho_{2}\left|=\right|\rho_{3}\left|=\cdots =\right|\rho_{n}|=\sqrt{\frac{1}{\left(n-1\right)}(2B-{\left(\frac{2{(}^{m}SO\left(G\right))}{n}\right)}^{2}}).$$

This completes the proof of Part (i). Next, we prove Part (ii). Since *G* contains no isolated vertex, according to Proposition 4, we have $\rho_{1}\geq \sqrt{\frac{2B}{n}}$, which gives $F\left(\rho_{1}\right)\leq F\left(\sqrt{\frac{2B}{n}}\;\right)$ (where *F* is defined via (10)), and hence we have

$$E_{MS}\left(G\right)\leq \sqrt{\frac{2B}{n}}+\sqrt{\left(n-1\right)\left(2B-{\left(\sqrt{\frac{2B}{n}}\right)}^{2}\right)}=\sqrt{2nB}.$$

Note that the equation $E_{MS}\left(G\right)=\sqrt{2nB}$ holds if and only if $\rho_{1}=\sqrt{\frac{2B}{n}}$ and $|\rho_{2}\left|=\right|\rho_{3}\left|=\cdots =\right|\rho_{n}|=\sqrt{\frac{2B}{n}}$, which holds if and only if $G≅\frac{n}{2}K_{2}.$ □

Our next upper bound on $E_{MS}$ is a consequence of a result credited to Filipovski and Jajcay [20].

**Theorem** **3.** *If G is a graph of the order n and t is a positive integer such that $\rho_{t}$ is positive, then*

(12) $$E_{MS}\left(G\right)\leq \sqrt{2Bn-\frac{2n}{B}{\left(\rho_{1}^{2}+\rho_{2}^{2}+\cdots +\rho_{t}^{2}-B\right)}^{2}}\;.$$

**Proof.**  The proof is similar to that of Theorem 5 of [20], and hence it is omitted here. □

The following result gives a lower bound on $E_{MS}$ in terms of the topological index B.

**Theorem** **4.** *If G is a connected graph of the order n, where n≥3, then*

$$E_{MS}\left(G\right)\geq 2\sqrt{B},$$

*with equality holding if and only if G is a complete bipartite graph.*

**Proof.**  Since

$$\sum_{i=1}^{n}\rho_{i}^{2}=-2\sum_{1\leq i<j\leq n}\rho_{i}\rho_{j},$$

we have

$$\begin{array}{cc} E_{MS}^{2}\left(G\right)=\sum_{i=1}^{n}\rho_{i}^{2}+2\sum_{1\leq i<j\leq n}|\rho_{i}\left|\right|\rho_{j}| & \geq \sum_{i=1}^{n}\rho_{i}^{2}+2\left|\sum_{1\leq i<j\leq n}\rho_{i}\rho_{j}\right|\\ \; & =2\sum_{i=1}^{n}\rho_{i}^{2}=4B, \end{array}$$

where the equation $E_{MS}^{2}\left(G\right)=4B$ holds if and only if

$$\sum_{1\leq i<j\leq n}|\rho_{i}\left|\right|\rho_{j}|=\left|\sum_{1\leq i<j\leq n}\rho_{i}\rho_{j}\right|,$$

which is possible if and only if $\rho_{1}=-\rho_{n}$ and $\rho_{2}=\rho_{3}=\cdots =\rho_{n-1}=0.$ Thus, *G* has three distinct modified Sombor eigenvalues and hence, according to Proposition 1.3.3 of [40], the diameter of *G* must be two. Moreover, we note that the modified Sombor spectrum of *G* is symmetric toward the origin, so it is verified that *G* is bipartite (see Lemma 2.12 of [22]). Consequently, it follows that *G* is a complete bipartite graph (see Theorem 2.1 of [41] and Corollary 3.8 of [43]). □

The next proposition is an immediate consequence of the equality case of Theorem 4, Proposition 1.3.3 of [40], and Theorem 2.1 of [41].

**Proposition** **7.** *Let G be a connected bipartite graph. The graph G has three distinct modified Sombor eigenvalues if and only if G is a complete bipartite graph.*

Next, we have a consequence of Theorems 3 and 4.

**Corollary** **2.** *Let G be a connected graph with exactly one positive modified Sombor eigenvalue. Then*

(13) $$E_{MS}\left(G\right)\leq 2\sqrt{B+B\sqrt{\frac{n-2}{n}}},$$

*with equality if and only if $G≅K_{2}.$*

**Proof.**  From Theorem 4, it follows that

(14) $$E_{MS}{\left(G\right)}^{2}\geq 4B,$$

with equality if and only if $G≅K_{a,n-a}.$ Moreover, since *G* has exactly one positive modified Sombor eigenvalue, Theorem 3 yields

(15) $$E_{MS}\left(G\right)\leq \sqrt{2nB-\frac{2n}{B}{\left(\rho_{1}^{2}-B\right)}^{2}}.$$

According to (14) and (15), we obtain

$$4B\leq 2nB-\frac{2n}{B}{\left(\rho_{1}^{2}-B\right)}^{2},$$

which implies that

(16) $$\rho_{1}\leq \sqrt{B+B\sqrt{\frac{n-2}{2}}}.$$

Therefore,

$$E_{MS}\left(G\right)=2\rho_{1}\leq 2\sqrt{B+B\sqrt{\frac{n-2}{2}}},$$

where the inequality sign becomes an equality sign if and only if *G* is $K_{2}.$ □

**Remark** **2.** *By Theorem 2,*

$${\left(E_{MS}\left(G\right)\right)}^{2}\leq 2nB,$$

*with equality if and only if $G≅\frac{n}{2}K_{2}.$ Moreover, Inequality (13) gives*

$${\left(E_{MS}\left(G\right)\right)}^{2}\leq 4B\left(1+1\sqrt{\frac{n-2}{n}}\right).$$

*The inequality*

$$4B\left(1+1\sqrt{\frac{n-2}{n}}\right)\leq 2nB$$

*holds whenever*

(17) $$n^{2}\left(n-2\right)>4,$$

*which holds for n≥3. Thus, for the graphs that have only one positive modified Sombor eigenvalue, the bound (13) is better than the second bound given in Theorem 2.*

The following result gives a lower bound on $E_{MS}$ in terms of $\rho_{n}$ and B.

**Theorem** **5.** *If G is a connected non-trivial graph of the order n, then*

(18) $$E_{MS}\left(G\right)\geq \left|\rho_{n}\right|+\sqrt{4B-3\rho_{n}^{2}},$$

*where the equality holds if and only if G is either the complete bipartite or the complete tripartite graph.*

**Proof.**  Since the trace of $A_{MS}\left(G\right)$ is zero, we have

$$\begin{array}{c} \rho_{n}^{2}={\left(\sum_{i=1}^{n-1}\rho_{i}\right)}^{2}=\sum_{i=1}^{n-1}\rho_{i}^{2}+2\sum_{1\leq i<j\leq n-1}\rho_{i}\rho_{j}, \end{array}$$

and

$${\left(\sum_{i=1}^{n-1}\left|\rho_{i}\right|\right)}^{2}=\sum_{i=1}^{n-1}\rho_{i}^{2}+2\sum_{1\leq i<j\leq n-1}|\rho_{i}\left|\cdot \right|\rho_{j}|.$$

Since $\rho_{n}^{2}\leq \frac{1}{2}\sum_{i=1}^{n-1}\rho_{i}^{2}$, we have

(19) $$\begin{array}{cc} {\left(E_{MS}\left(G\right)-\left|\rho_{n}\right|\right)}^{2}={\left(\sum_{i=1}^{n-1}\left|\rho_{i}\right|\right)}^{2} & =\sum_{i=1}^{n-1}\rho_{i}^{2}+2\sum_{1\leq i<j\leq n-1}|\rho_{i}\left|\cdot \right|\rho_{j}|\\ \; & \geq \sum_{i=1}^{n-1}\rho_{i}^{2}+\left|2\sum_{1\leq i<j\leq n-1}\rho_{i}\cdot \rho_{j}\right|\\ \; & =\sum_{i=1}^{n-1}\rho_{i}^{2}+\left|\rho_{n}^{2}-\sum_{i=1}^{n-1}\rho_{i}^{2}\right|\\ \; & =2\sum_{i=1}^{n}\rho_{i}^{2}-3\rho_{n}^{2}=4B-3\rho_{n}^{2}. \end{array}$$

Thus, we obtain

$$E_{MS}\left(G\right)\geq \left|\rho_{n}\right|+\sqrt{4B-3\rho_{n}^{2}}.$$

Equality holds in (19) if and only if

$$\sum_{1\leq i<j\leq n-1}|\rho_{i}\left|\cdot \right|\rho_{j}|=\left|\sum_{1\leq i<j\leq n-1}\rho_{i}\cdot \rho_{j}\right|.$$

One such possibility is $\rho_{2}=\rho_{3}=\cdots =\rho_{n-1}=0,$ and it follows that $\rho_{1}=-\rho_{n},$ since $Tr(A_{MS}\left(G\right))=0$. This implies that the modified Sombor spectrum of *G* is symmetric toward its origin, i.e., *G* is bipartite, and, according to Proposition 7, *G* is the complete bipartite graph. Conversely, $E_{MS}\left(K_{a,n-a}\right)=2\rho_{1}=\left|\rho_{n}\right|+\sqrt{\rho_{1}^{2}}.$ The second possibility is that the modified Sombor spectrum of *G* is

(20) $$\{\rho_{1},\underset{n-3}{\underset{︸}{0,0,\ldots ,0,0}},-\rho_{n-1},-\rho_{n}\},$$

and, in this case,

$$\begin{array}{cc} 2\rho_{1}\rho_{n-1} & =2\sum_{1\leq i<j\leq n-1}|\rho_{i}\left|\cdot \right|\rho_{j}|=\left|2\sum_{1\leq i<j\leq n-1}\rho_{i}\cdot \rho_{j}\right|\\ \; & =|2\rho_{n}\left(-\rho_{n-1}\right)|=2\rho_{1}\rho_{n-1}. \end{array}$$

Next, we show that the spectrum given in (20) is the modified Sombor spectrum of the complete tripartite graph. Let

$$\left\{u_{1},u_{2},\ldots ,u_{a},v_{1},v_{2},\ldots ,v_{b},w_{1},w_{2},\ldots ,w_{c}\right\}$$

be the vertex labeling of the tripartite graph $G≅K_{a,b,c},\left(a+b+c=n\right).$ Under this labeling, $d_{u_{1}}=d_{u_{2}}=\cdots =d_{u_{a}}=b+c=n-a,d_{v_{1}}=d_{v_{2}}=\cdots =d_{v_{a}}=a+c=n-b,$ and $d_{w_{1}}=d_{w_{2}}=\cdots =d_{w_{a}}=a+b=n-c,$ and the modified Sombor matrix of *G* can be written as

(21) $$A_{MS}\left(G\right)=\left(\begin{array}{ccc} O_{a\times a} & \frac{1}{\sqrt{d_{1}^{2}+d_{2}^{2}}}J_{a\times b} & \frac{1}{\sqrt{d_{1}^{2}+d_{3}^{2}}}J_{a\times c}\\ \frac{1}{\sqrt{d_{1}^{2}+d_{2}^{2}}}J_{b\times a} & O_{b\times b} & \frac{1}{\sqrt{d_{2}^{2}+d_{3}^{2}}}J_{b\times c}\\ \frac{1}{\sqrt{d_{1}^{2}+d_{2}^{2}}}J_{c\times a} & \frac{1}{\sqrt{d_{2}^{2}+d_{3}^{2}}}J_{c\times b} & O_{c\times c} \end{array}\right),$$

where O is the zero matrix, and *J* is the matrix of all ones. For i=2,3…,a;j=2,3,…,b; and k=2,3,…,c, consider the following vectors:

$$\begin{array}{cc} X_{i-1} & =(-1,x_{i2},x_{i3},\ldots ,x_{ia},\underset{n-a}{\underset{︸}{0,0,\ldots ,0,0)}}\;\mathrm{where}\;x_{il}=\left\{\begin{array}{cc} 1 & \mathrm{if}\;i=l\\ 0 & \mathrm{otherwise}, \end{array}\right.\\ Y_{i-1} & =(\underset{a}{\underset{︸}{0,0,\ldots ,0,0}},-1,y_{j2},x_{j3},\ldots ,x_{jb},\underset{c}{\underset{︸}{0,0,\ldots ,0,0)}}\;\mathrm{where}\;y_{il}=\left\{\begin{array}{cc} 1 & \mathrm{if}\;j=l\\ 0 & \mathrm{otherwise}, \end{array}\right.\\ Z_{i-1} & =(\underset{n-c}{\underset{︸}{0,0,\ldots ,0,0)}},-1,z_{k2},z_{k3},\ldots ,z_{kc}\;\mathrm{where}\;z_{il}=\left\{\begin{array}{cc} 1 & \mathrm{if}\;k=l\\ 0 & \mathrm{otherwise}. \end{array}\right. \end{array}$$

Clearly,

$$\begin{array}{cc} AX_{1} & =(\underset{a}{\underset{︸}{0,0,\ldots ,0}},\underset{b}{\underset{︸}{\frac{1}{\sqrt{d_{1}^{2}+d_{2}^{2}}}-\frac{1}{\sqrt{d_{1}^{2}+d_{2}^{2}}},\frac{1}{\sqrt{d_{1}^{2}+d_{2}^{2}}}-\frac{1}{\sqrt{d_{1}^{2}+d_{2}^{2}}},\ldots ,\frac{1}{\sqrt{d_{1}^{2}+d_{2}^{2}}}-\frac{1}{\sqrt{d_{1}^{2}+d_{2}^{2}}}}},\\ \; & \;\underset{c}{\underset{︸}{\frac{1}{\sqrt{d_{1}^{2}+d_{3}^{2}}}-\frac{1}{\sqrt{d_{1}^{2}+d_{3}^{2}}},\frac{1}{\sqrt{d_{1}^{2}+d_{3}^{2}}}-\frac{1}{\sqrt{d_{1}^{2}+d_{3}^{2}}},\ldots ,\frac{1}{\sqrt{d_{1}^{2}+d_{3}^{2}}}-\frac{1}{\sqrt{d_{1}^{2}+d_{3}^{2}}}}})=0X_{1}. \end{array}$$

Similarly, $X_{1},X_{2},\ldots ,X_{a-1},Y_{1},Y_{2},\ldots ,Y_{b-1},$ and $Z_{1},Z_{2},\ldots ,Z_{c-1}$ are the eigenvectors corresponding to the eigenvalue 0. Thus, 0 is the modified Sombor eigenvalue of *G* with the multiplicity a+b+c−3. The remaining three modified Sombor eigenvalues of *G* are the eigenvalues of the following equitable quotient matrix (see Section 2.3 of [40])

(22) $$A_{Q}=\left(\begin{array}{ccc} 0 & \frac{b}{\sqrt{d_{1}^{2}+d_{2}^{2}}} & \frac{c}{\sqrt{d_{1}^{2}+d_{3}^{2}}}\\ \frac{a}{\sqrt{d_{1}^{2}+d_{2}^{2}}} & 0 & \frac{c}{\sqrt{d_{2}^{2}+d_{3}^{2}}}\\ \frac{a}{\sqrt{d_{1}^{2}+d_{2}^{2}}} & \frac{b}{\sqrt{d_{2}^{2}+d_{3}^{2}}} & 0 \end{array}\right).$$

The determinant of above matrix is

$$\frac{2abc}{\sqrt{4a^{2}+4ab+4ac+4b^{2}+4bc+4c^{2}}},$$

which is certainly positive. Moreover, since $A_{Q}$ has the positive determinant and $Tr(A_{Q})=0,$ the matrix $A_{Q}$ has one positive eigenvalue $\rho_{1}$ (according to the Perron–Frobenius theorem) and two negative eigenvalues $\rho_{n-1},\rho_{n}.$ Thus, the desired equality holds if and only if *G* is the complete tripartite graph. □

The graph obtained from $K_{\omega }$ and $P_{l}$ by adding an edge between any vertex of $K_{\omega }$ and an end vertex of $P_{l}$ is denoted by $PK_{\omega ,l}$ is known as a *path complete graph* or *kite graph*. The *pineapple* graph P(ω,n−ω) is the graph obtained from $K_{\omega }$ by attaching n−ω pendent vertices to any vertex of $K_{\omega }.$

Table 2 and Table 3 give the numerical values of the bounds on the modified Sombor energy obtained in the present article.

From Table 2, for graphs with one positive modified Sombor eigenvalue, Corollary 2 gives a better upper bound, and, for general graphs, Theorem 2 (i) (the Koolen–Moulton-type bound) gives a better upper bound. From Table 3 and with computational experiments, we observe that Theorem 3.6 of Huang and Liu [16] gives a better lower bound for graphs with a large diameter. Alternatively, for graphs with few positive modified Sombor eigenvalues and a small diameter, along with large independence and clique numbers, other lower bounds in this article are better than that of Theorem 3.6 in [16].

Next, we determine an inequality between $E_{MS}$, E, and $E_{SO}$ for the case of the path graph $P_{n}$ of order *n*, where n≥4. For this, we need the following result:

**Lemma** **1** ([44]). *If $U_{1}$ and $U_{2}$ are square matrices of the order n, then*

$$\sum_{i=1}^{n}\sigma_{i}\left(U_{1}+U_{2}\right)\leq \sum_{i=1}^{n}\sigma_{i}\left(U_{1}\right)+\sum_{i=1}^{n}\sigma_{i}\left(U_{2}\right),$$

*with equality if and only if there exists an orthogonal matrix M, such that $MU_{1}$ and $MU_{2}$ are both positive semi-definite.*

**Proposition** **8.** *For n≥4, if $P_{n}$ is the path graph with n vertices, then*

$$E_{MS}\left(P_{n}\right)\leq E\left(P_{n}\right)\leq E_{SO}\left(P_{n}\right)$$

**Proof.**  The modified Sombor matrix of $P_{n}$ can be written as

$$\begin{array}{c} A_{MS}\left(P_{n}\right)=\frac{1}{2\sqrt{2}}A\left(P_{n}\right)+R \end{array}$$

where

$$R=\left(\begin{array}{ccccccc} 0 & \frac{1}{\sqrt{5}}-\frac{1}{2\sqrt{2}} & 0 & \ldots & 0 & 0 & 0\\ \frac{1}{\sqrt{5}}-\frac{1}{2\sqrt{2}} & 0 & 0 & \ldots & 0 & 0 & 0\\ 0 & 0 & 0 & \ldots & 0 & 0 & 0\\ \vdots & \vdots & \vdots & \ddots & \vdots & \vdots & \vdots \\ 0 & 0 & 0 & \ldots & 0 & 0 & 0\\ 0 & 0 & 0 & \ldots & 0 & 0 & \frac{1}{\sqrt{5}}-\frac{1}{2\sqrt{2}}\\ 0 & 0 & 0 & \ldots & 0 & \frac{1}{\sqrt{5}}-\frac{1}{2\sqrt{2}} & 0 \end{array}\right).$$

We note that $\frac{1}{20}\left(4\sqrt{5}-5\sqrt{2}\right)$ is an eigenvalue of *R* with corresponding eigenvectors $\left(0,0,\ldots ,0,1,1\right)$ and $\left(1,1,0,\ldots ,0,0\right)$. Similarly, $\frac{1}{20}\left(5\sqrt{2}-4\sqrt{5}\right)$ is another eigenvalue of *R* with corresponding eigenvectors $\left(-1,1,0,\ldots ,0,0\right)$ and $\left(0,0,\ldots ,0,-1,1\right)$. Moreover, 0 is also an eigenvalue of *R* with the multiplicity n−4. The absolute sum of the eigenvalues of the symmetric matrix *R* equals $\frac{1}{5}\left(4\sqrt{5}-5\sqrt{2}\right)$, i.e.,

$$E\left(R\right)=\frac{1}{5}\left(4\sqrt{5}-5\sqrt{2}\right)\approx 0.374641.$$

Therefore, according to Lemma 1, we have

(23) $$E_{MS}\left(P_{n}\right)\leq \frac{1}{2\sqrt{2}}E\left(P_{n}\right)+E\left(R\right)\approx \frac{1}{2\sqrt{2}}E\left(P_{n}\right)+0.374641.$$

Similarly, we have

$$\begin{array}{c} A_{S}\left(P_{n}\right)=2\sqrt{2}A\left(P_{n}\right)+B, \end{array}$$

where

$$B=\left(\begin{array}{cccccc} 0 & \sqrt{5}-2\sqrt{2} & 0 & \ldots & 0 & 0\\ \sqrt{5}-2\sqrt{2} & 0 & 0 & \ldots & 0 & 0\\ 0 & 0 & 0 & \ldots & 0 & 0\\ 0 & 0 & 0 & \ldots & 0 & 0\\ \vdots & \vdots & \vdots & \ddots & \vdots & \vdots \\ 0 & 0 & 0 & \ldots & 0 & 0\\ 0 & 0 & 0 & \ldots & 0 & \sqrt{5}-2\sqrt{2}\\ 0 & 0 & 0 & \ldots & \sqrt{5}-2\sqrt{2} & 0 \end{array}\right).$$

The spectrum of *B* is $\left\{{\left(\sqrt{5}-2\sqrt{2}\right)}^{\left[2\right]},{\left(2\sqrt{2}-\sqrt{5}\right)}^{\left[2\right]},0^{\left[n-4\right]}\right\}.$ Therefore, according to Lemma 1, we have

$$\begin{array}{c} E_{SO}\left(P_{n}\right)\leq 2\sqrt{2}E\left(P_{n}\right)+E\left(B\right)\approx 2\sqrt{2}E\left(P_{n}\right)+2.36944. \end{array}$$

Thus, we have

$$\frac{1}{2\sqrt{2}}E\left(P_{n}\right)+0.374641\overset{<}{\approx }E\left(P_{n}\right)\overset{<}{\approx }2\sqrt{2}E\left(P_{n}\right)+2.36944.$$

□

**Example** **1.** *The modified Sombor energy of $P_{26}$ up to four decimal places is 11.7798, the energy of $P_{26}$ is 32.3969, and the Sombor energy of $P_{28}$ is 89.6643. Alternatively, according to Proposition 8, the upper bound for the modified Sombor energy of $P_{19}$ is 22.9081, the upper bound for the Sombor energy is 94.0017, and the bounds for the energy are $22.9081\leq E(P_{26})\leq 94.0017.$*

## 3. Modified Sombor Equienergetic Graphs and Chemical Applicability of the Modified Sombor Index/Energy

**Figure 2:**
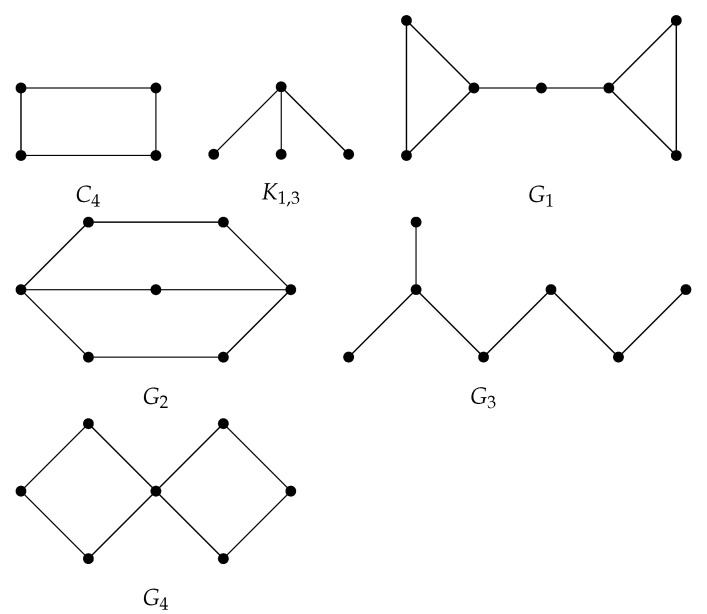
Three pairs of equienergetic chemical graphs of the order of at most 7, namely $\left\{C_{4},\;K_{1,3}\right\}$, $\left\{G_{1},\;G_{2}\right\}$, and $\left\{G_{3},\;G_{4}\right\}$. Among these three pairs, $\left\{C_{4},\;K_{1,3}\right\}$ and $\left\{G_{3},G_{4}\right\}$ are neither Sombor equienergetic graphs nor modified Sombor equienergetic graphs.

Two non-isomorphic graphs $G_{1}$ and $G_{2}$ of the order *n* that have the same energy/Sombor energy/modified Sombor energy are known as equienergetic graphs/Sombor equienergetic graphs/modified Sombor equienergetic graphs, respectively. By using computer programs (Mathematica and AutographiX), we found that there exists only one pair of Sombor equienergetic graphs and only one pair of modified Sombor equienergetic graphs among all chemical graphs of the order of at most seven. However, there are exactly three pairs of equienergetic graphs among all chemical graphs of the order of at most seven. Two pairs of equienergetic graphs, namely $\left\{C_{4},\;K_{1,3}\right\}$ and $\left\{G_{3},\;G_{4}\right\}$ (see Figure 2 and Table 4), are neither Sombor equienergetic graphs nor modified Sombor equienergetic graphs. This gives the insight that Sombor equienergetic graphs and modified Sombor equienergetic graphs are rare in comparison to equienergetic graphs.

Next, we carry the (linear, logarithmic, and quadratic) regression analyses for the Sombor index, modified Sombor index, Sombor energy, and modified Sombor energy on the class of all chemical graphs of the order of at most 7 to check their predictive abilities for the case of boiling points. The data on the boiling points for the aforementioned chemical graphs are taken from [45], and the other parameters are calculated by AutographiX [46]. Table 5 gives the correlation of the boiling points (Bp) with each of the following topological indices: the Sombor index, Sombor energy, modified Sombor index, and modified Sombor energy.

**Figure 3:**
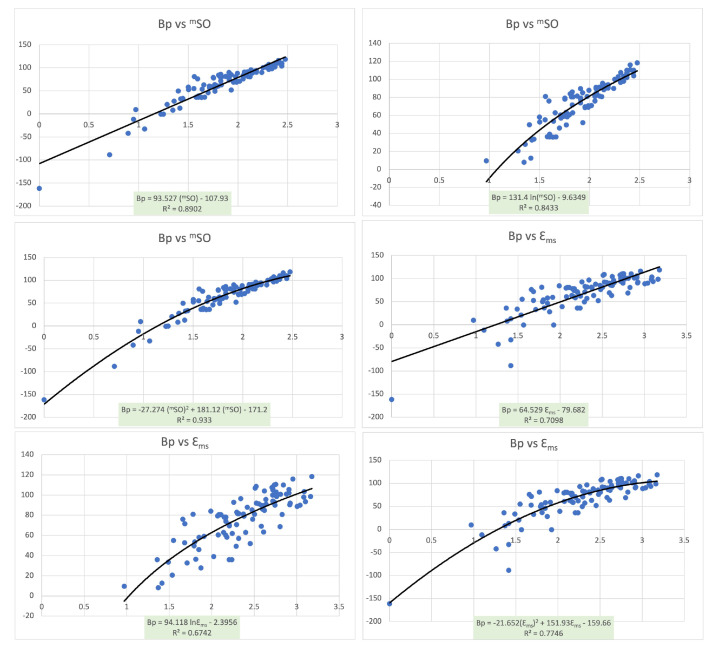
The scattering of Bp (boiling points) with each of the topological indices ${}^{m}SO$ and $E_{MS}$ for the linear, logarithmic, and quadratic regressions along with the regression equations and $R^{2}$ (coefficient of determination).

Table 5 suggests that the modified Sombor index is better correlated with the boiling points than all of the other three considered topological indices. Moreover, the modified Sombor energy is the second-best predictor for the boiling points among the considered indices. The scattering of Bp (boiling points) with each of the topological indices ${}^{m}SO$ and $E_{MS}$ for the linear, logarithmic, and quadratic regressions along with the regression equations and $R^{2}$ (coefficient of determination), are shown in Figure 3.

Figure 3 and Table 6 indicate that the modified Sombor index ${}^{m}SO$ has a better coefficient of determination with the boiling points than that of the Sombor index SO in all three regressions. Similarly, the coefficient of determination of the modified Sombor energy $E_{MS}$ with the boiling points is better than that of the Sombor energy $E_{SO}$ in all three regressions.

## 4. Conclusions

Every weighted adjacency matrix has its own importance. The following facts seem to be interesting to note about some particular weighted adjacency matrices:

- The energy of a graph cannot be an odd integer (Bapat and Pati [36]).
- The arithmetic–geometric energy of a graph can be any integer greater than one (Zheng, Tian and Cui [37]).
- The modified Sombor energy of every regular complete multipartite is $\sqrt{2}$. Thereby, we obtain a large family of modified Sombor equienergetic graphs (see Corollary 1).
- The modified Sombor spectral radius of every regular graph is $\frac{1}{\sqrt{2}}$ (see Proposition 2).

Moreover, we remark that there exists only one pair of Sombor equienergetic graphs and only one pair of modified Sombor equienergetic graphs among all chemical graphs of the order of at most seven. This gives the insight that Sombor equienergetic graphs and modified Sombor equienergetic graphs are rare in comparison to equienergetic graphs (there exists exactly three pairs of equienergetic graphs among all chemical graphs of the order of at most seven). Furthermore, we remark that the modified Sombor index and modified Sombor energy give a better correlation than their corresponding classical versions with the boiling points of the chemical graphs of the order of at most seven; this provides motivation to further study the topological indices defined via the modified Sombor matrix.

The Sombor matrix (modified Sombor) and their corresponding indices are new topics of research both in mathematics and theoretical chemistry. All of the linear algebraic properties of these matrices have yet to be investigated, especially their spectral radii, energies, norms, Estrada indices, eigenvalue distributions, and, most importantly, the characterization of their extremal graphs. Similarly, the modified Sombor index is new, and all interesting properties are unknown, specifically in the chemical modeling of alkanes.

## Figures and Tables

**Table 1:** Table showing value of modified Sombor index for some graphs, numerical values of the bounds obtained in Propositions 1 and 3, and the different results of Huang and Liu [16].

Graph *G*	${}^{m}SO\left(G\right)$	Propositions 1	Propositions 3	Theorem 2.1	Corollary 2.3	Corollary 2.4
$P_{14}$	4.78351	4.89003	4.80364	4.88908	6.05761	4.94975
$CS_{5,3}$	2.75387	2.78203	2.76018	2.80299	4.49642	2.82843
DS(16, 3, 4)	4.70027	5.01065	5.06802	5.30254	6.78914	5.65685
$S_{5}^{+}$	1.31329	1.66039	1.36239	2.18222	2.12132	2.12132
CG10	2.82008	3.22145	2.84684	3.22319	4.66404	3.53553
Graph *G*	${}^{m}SO\left(G\right)$	Theorem 2.7	Cor. 2.8	Theorem 2.16	Corollary 2.18	Corollary 2.21
$P_{14}$	4.78351	9.19239	9.89949	8.13909	9.1115	5.0104
$CS_{5,3}$	2.75387	3.53553	3.9598	7.38738	3.50586	2.782
DS(16, 3, 4)	4.70027	10.6060	28.28443	9.01171	9.92488	5.14324
$S_{5}^{+}$	1.31329	4.24264	10.6066	3.44814	3.566	1.85567
CG10	2.82008	10.6066	17.6777	6.82982	9.96694	3.71687

**Table 2:** Modified Sombor energy and the approximate values of the upper bounds obtained in this article.

*G*	$E_{\mathrm{SO}}\left(G\right)$	Theorem 2 (i)	Theorem 2 (ii)	Theorem 3	Corollary 2
$P_{14}$	6.38433	7.01418	7.04982	7.04982	NA
$CS_{5,3}$	1.39101	1.66237	2.20814	2.56084	1.50819
$K_{3,4,5}$	1.40331	2.39582	3.00054	3.14352	1.69421
$PK_{3,9}$	5.29907	5.81504	5.8599	5.86874	NA
P(4,3)	1.60161	2.03389	2.098059	2.19134	NA
$K_{12}$	1.41422	1.41422	2.55841	3.33034	1.44457

**Table 3:** Modified Sombor energy and the approximate values of the lower bounds obtained in the present paper and Theorem 3.6 of [16].

*G*	$E_{\mathrm{SO}}\left(G\right)$	Theorem 1 (i)	Theorem 1 (ii)	Theorem 4	Theorem 5	Theorem 3.6 [16]
$P_{14}$	6.38433	1.36672	1.00712	2.66458	3.0726	5.80258
$CS_{5,3}$	1.39101	1.37694	0.552036	1.10407	1.27336	0.967999
$K_{3,4,5}$	1.40331	1.39786	0.50009	1.22496	1.40331	1.22496
$PK_{3,9}$	5.29907	1.36923	0.976651	2.3923	2.76237	4.77683
P(4, 3)	1.60161	0.94144	0.599442	1.12145	1.29488	1.25262
$K_{12}$	1.41422	1.41421	0.426402	1.04447	1.1028	0.805388

**Table 4:** Approximate values of the energy, Sombor energy, and modified Sombor energy of the graphs depicted in Figure 2.

Energy	$C_{4}$	$K_{1,3}$	$G_{1}$	$G_{2}$	$G_{3}$	$G_{4}$
E(G)	4	4	9.62721	9.62721	7.72741	7.72741
$E_{SO}\left(G\right)$	11.3137	16.4924	32.3713	32.3713	22.3639	27.5959
$E_{MS}\left(G\right)$	1.41421	0.970143	2.90798	2.90798	2.74436	2.34164

**Table 5:** Correlation of the boiling points (Bp) with each of the following topological indices for the case of all chemical graphs of the order of at most 7: Sombor index, Sombor energy, modified Sombor index, and modified Sombor energy.

Bpvs.SO(G)	$\mathrm{Bp}\;\mathrm{vs}.\;E_{\mathrm{SO}}\left(G\right)$	$\mathrm{Bp}\;\mathrm{vs}.{\;}^{m}\mathrm{SO}\left(G\right)$	$\mathrm{Bp}\;\mathrm{vs}.\;E_{\mathrm{MS}}\left(G\right)$
0.720862158	0.809447751	0.943525603	0.842522597

**Table 6:** The coefficient of determination of the boiling points with the topological indices $SO,\;E_{SO}$, ${}^{m}SO$, and $E_{MS}$ for the linear, logarithmic, and quadratic regressions.

Topological Index	Linear	Logarithmic	Quadratic
SO	0.5196	0.3069	0.7136
$E_{SO}$	0.6552	0.4624	0.8119
${}^{m}SO$	0.8902	0.8433	0.933
$E_{MS}$	0.7098	0.6742	0.7746

## Data Availability

The data used to support the findings of the results are included within the article.
